# New stable anchor protein and peptide linker suitable for successful spore surface display in *B. subtilis*

**DOI:** 10.1186/1475-2859-12-22

**Published:** 2013-02-28

**Authors:** Krzysztof Hinc, Adam Iwanicki, Michał Obuchowski

**Affiliations:** 1 Laboratory of Molecular Bacteriology, Intercollegiate Faculty of Biotechnology UG & MUG, Medical University of Gdańsk, Dębinki 1, Gdańsk 80-211, Poland

## Abstract

**Background:**

In last decade spores have been successfully used as a surface display platform. Various peptides or proteins were displayed this way as functional enzymes or antigens. Nearly all attempts involved use of three coat proteins: CotB, CotC or CotG. Increasing knowledge of the structure of the spore coat allowed us to propose the use of other proteins whose localization in the spore envelope has been determined. We also propose the application of a new linker suitable for building fusion proteins.

**Results:**

We show that a member of the outer coat, CotZ, is a good candidate as a new anchor protein useful in spore surface display. This protein allows use of relatively large passenger proteins and their efficient display on the spore surface. Analysis by Western- and dot-blotting, combined with immunofluorescence microscopy, allowed us to estimate the number of displayed fusion proteins molecules as 1.4 × 10^2^ per spore. In addition, we present data indicating that the use of a peptide linker, which forms a stable α-helix, may greatly improve the display of anchored proteins on the spore surface.

**Conclusion:**

CotZ can be used as an efficient anchor protein in the outer spore coat. Its localisation in the coat crust layer should guarantee surface display of passenger proteins. Moreover, a CotZ based fusion can tolerate relatively large passenger proteins for efficient spore surface display. In addition, to the properties of both the anchor and passenger proteins, an important issue is the nature of the linker. Here we present evidence that the linker, which forms a stable α-helix, may be crucial for successful display.

## Introduction

Cell surface display is a powerful method successfully used in research as well as in applied applications. In the last decade bacterial spores drew the attention of researchers as useful bio-particles suitable for surface display. Spores can be relatively easily modified by introducing appropriate changes in the chromosomal DNA of the vegetative cells. However, it is important, that such modification does not affect the ability of the dormant spore to survive harsh conditions that might be encountered, for example, in the human gut.

*Bacillus subtilis* is the most studied spore-forming bacterium, its spore has a central core that contains chromosomal DNA and cellular proteins necessary for the return to vegetative growth. The core is encased by thick layer of modified peptidoglycan named the cortex. The cortex is surrounded by a protein multilayer named the coat. The spore coat consists of over 70 proteins, which become organised in several layers during spore development [1,2], for review see [3]. Importantly, *B. subtilis* is a non-pathogenic bacterium widely used as probiotic for human and animal consumption [4]. These attributes make *B. subtilis* spores an attractive vehicle for heterologous protein surface display [5-8]. So far successful heterologous protein display was reported for the use of three outer and one inner spore coat proteins as carriers: CotB, CotC, CotG and OxdD respectively [9-14]. The selection of these proteins as carriers was based not only on their location but also their relative abundance. However, successful heterologous spore surface display is determined by several factors: the localisation of the anchor protein, its properties, the size and properties of the displayed passenger protein and how this is linked to the spore coat protein. It was shown previously, that the same passenger protein might give a variety of results when displayed will different coat proteins [10]. Detailed examination of coat proteins localisation revealed that at least four spatially distinct layers surrounding the spore [15]. Recently, new members, CotZ and CgeA of the outermost layer (crust) of spore coat were identified. The CotZ together with CotY and CotZ are morphogenetic proteins of the spore crust, which mostly consists of CotW protein [3,16]. The position of these proteins is close to the spore coat surface and their accessibility for antibodies suggests that they can be good candidates for new anchor proteins for spore surface display. Moreover, the lack of the crust layer has no impact on resistance properties of spores tested in laboratory conditions [10]. In addition, the method of linking (direct or through the linker) plays an important role in successful display. Unfortunately, currently it is nearly impossible to predict if direct linkage of the coat protein to the passenger will be successful or that any linker is necessary. Usually this requirement is checked experimentally by analysis of the expression efficiency of the particular fusion protein.

Here we report the successful use of CotZ as a novel anchor for heterologous proteins suitable for efficient spore surface display. In order to compare the efficiency of spore display we used again as passenger, the urease subunit of *Helicobacter acinonychis*[10], an animal pathogen closely related to *H. pylori.* In addition, we show the importance of the secondary structure of the linker used for building the fusion protein.

## Results

### Construction and integration of the fusion gene *cotZ-ureA* into chromosome

**Figure 1:**
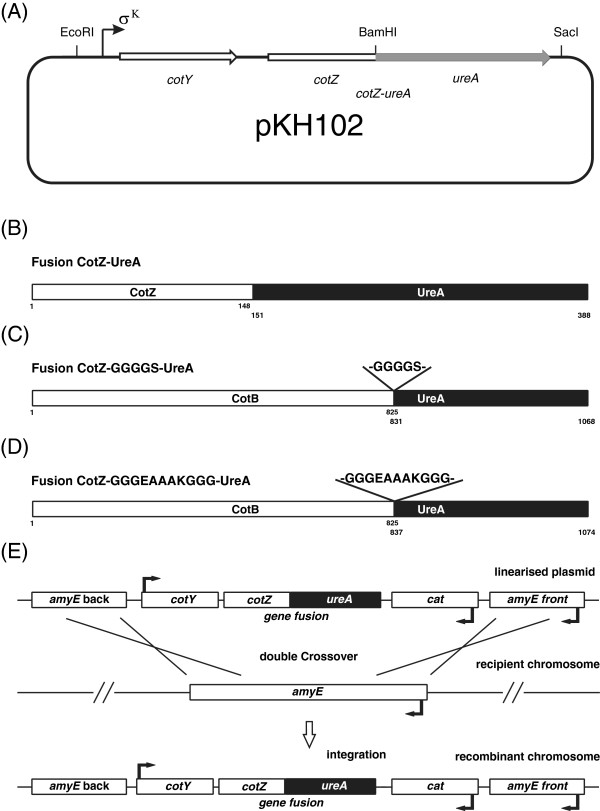
**Schematic steps of building strains carrying fusion genes. **A fragment containing the operon *cotYZ* was amplified and cloned into the pDL vector. Next, fragment containing the entire *ureA* gene was amplified and cloned in frame with the *cotZ* gene resulting in plasmid pKH102 (panel **A**). As a result we obtained the fusion protein CotZ-UreA (panel **B**). Similarly the CotB-GGGGS-UreA (panel **C**) and CotB-GGGEAAAKGGG-UreA (panel **D**) fusions were obtained. All numbers indicate amino acid residues. Next, pKH102 after linearization is integrated into the *B. subtilis* chromosome after a double crossing-over event (panel **E**), generating strain BKH102, which contains the *cotZ-ureA* fusion gene under control of the sigma K promoter.

The region containing the *cotYZ* genes, together with their promoter, was PCR amplified and finally, as described in Methods, cloned in frame, at the 3’ end of the *cotZ* gene carried by plasmid pDL-CotYZ. This yielded plasmid pKH102 containing the *cotZ-ureA* fusion gene (see Figure 1A). The fusion gene contains full-length *cotZ* and *ureA* (shown in Figure 1B), as confirmed by sequencing. Finally, pKH102 was used to transform competent cells of *B. subtilis* strain 168 as indicated at Methods and selection for chloramphenicol-resistant (Cm^R^) clones, resulting from interruption of the non-essential *amyE* gene in the *B. subtilis* chromosome (Figure 1C). A correct clone was identified by PCR (see Methods) and named BKH102.

### Construction and integration of fusion gene *cotB-linker-ureA* into chromosome

Previously we built a fusion consisting of the major part of the CotB protein (amino acid residues 1 to 825) and a major part of UreA (amino acid residues 148 to 717). However, surface display was very inefficient [10]. A possible explanation was the burying of UreA in the spore coat due to its direct linking to CotB and we anticipated that the close connection between the partners should solve this problem. Two peptide linkers were tested: one without any secondary structure (−GGGGS-) or a second with the strong alpha-helix motif (−GGGEAAAKGGG-) [17]. These linkers were placed between the C-terminus of CotB and the N-terminus of UreA by using the appropriate sequence as primer for the distal part of the *cotB* gene (see Table 1). Both fusion genes were integrated into the *B. subtilis* chromosome in *amyE* locus, and individual transformant colonies were tested by PCR (not shown) to identify the required clones and named BKH119 (fusion CotB-GGGGS-UreA) and BKH124 (fusion CotB-GGGEAAAKGGG-UreA).

**Table 1:** Oligonucleotide list

**Name**	**Sequence (5’-3’)**	**Restriction site**
cotY-F	GCT TA**G GAT CC**A TGA TGA TGT GTA CGA TTG	*Bam*HI
cotZ-R	CGT AGC **GAA TTC** AGT TAT CAC TCT TGT CCT C	*Eco*RI
Linker-gggs-F	CCG **GAA TTC** ACG GAT TAG GCC GTT TGT CCT CAT GGA CCC GTA TAA AAA GAA TGA TAT TGA GCG TTT TGA CCG TGA G	*Eco*RI
Linker-gggs-R	CGC **GAG CTC** TGC GCG GCC GCT AGA GCC ACC GCC ACC GTA GGA TCC GGA TGA TTG ATC ATC TGA AGA TTT TAG TGA TCG TTT AGA TG	*SacI*
linker-gggeaaakggg-F	GCC TGT TAG **GAA TTC** CGC TCC AAT CTC TTT TTA CAA TAG AAT ATA TGG AAC CGA AAA TCA TGG CGA TGT ATG AAC GGA TTA GGC C	*Eco*RI
linker- gggeaaakggg- R	CGC **GGA TCC** TCC TCC ACC TTT CGC TGC TGC TTC TCC TCC ACC GGA TGA TTG ATC ATC TGA AG	*Bam*HI
ureA-F	GAG **GGA TCC** ATG AAA CTC ACC CCA AAA G	*Bam*HI
ureA-R	CGC **GAG CTC** TAG GGC CAT ACA TAG AAA C	*SacI*

In bold are the recognition sites for the restriction enzymes indicated in the table.

### Expression of fusion genes

**Figure 2:**
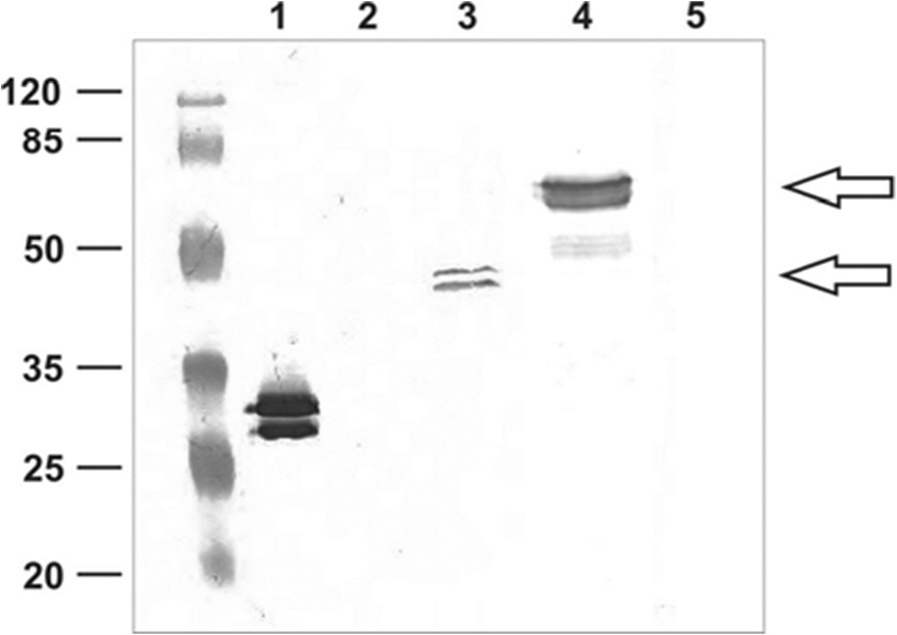
**Western blotting analysis of expression of the *cotZ-ureA *fusion gene. **The 168 and BKH102 (*amyE::cotZ-ureA*) strains were grown at 37°C with shaking for 24 h in Difco Sporulation medium as described elsewhere [18]. Spores were harvested and purified. Spore coats were extracted and subjected to SDS-PAGE, followed by Western blotting with anti-UreA antibodies. Lanes: 1 – purified UreA protein, 2 – spore coat proteins from spores of 168 strain, 3 – spore coat proteins from spores of strain BKH102 (fusion CotZ-UreA), 4 – spore coat proteins from spores of strain BKH124 (fusion CotB-GGGEAAAKGGG-UreA), 5 - spore coat proteins from spores of strain BKH119 (fusion CotB-GGGS-UreA). Each lane has the same protein load, 20 μg of total protein extract.

To verify expression of the fusion genes and localisation of their products we used a Western blotting approach with anti-UreA antibodies, against purified UreA, and spore coat extracts form strains 168, BKH102, BKH119 and BKH124 (Figure 2). We obtained a strong signal with pure UreA protein and extracts from spore coats of BKH102 (fusion CotZ-UreA) and BKH124 (fusion CotB-GGGEAAAKGGG-UreA). However, the fusion protein form the BKH119 (fusion CotB-GGGGS-UreA) was not detected in the coat protein extract as well as in the total protein extract of sporulation cells. The calculated molecular mass of CotZ-UreA and CotB-GGGEAAAKGGG-UreA fusion proteins is 42.8 kDa and 69.2 kDa respectively, which is consistent with the observed position of the bands. Post-translational processing of the UreA protein may cause the appearance of the double bands seen in Figure 2 with purified UreA and its fusion derivatives. For further analyses we selected the strains BKH102 (fusion CotZ-UreA) and BKH124 (fusion CotB-GGGEAAAKGGG-UreA).

### Surface display

**Figure 3:**
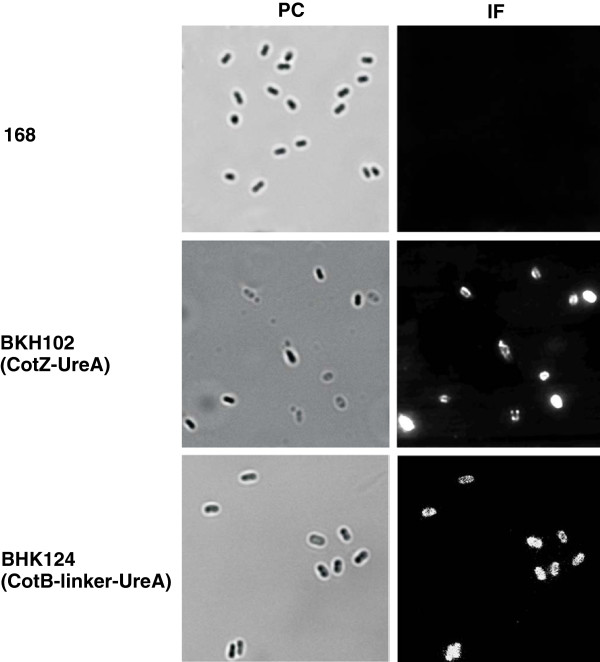
**Localisation of CotZ-UreA and CotB-GGGEAAAKGGG-UreA fusion proteins as assessed by immunofluorescence microscopy. **Purified, free spores of wild type strain 168, BKH102 (*cotZ-ureA*) and BKH124 (*cotB-*GGGEAAAKGG*-ureA*) were visualised by phase contrast (PC) and immunofluorescence (IF) microscopy. The spores were incubated with mouse anti-UreA antibodies, followed by anti-mouse IgG-Cy3 conjugates. The same exposure time was used for IF images.

In order to verify surface localisation of CotZ-UreA (BKH 102) and CotB-GGGEAAAKGGG-UreA (BKH124) fusion proteins dormant spores of wild type and recombinant strains were analysed by immunofluorescence microscopy with anti-UreA primary antibodies and anti-mouse IgG-Cy3 (Jackson ImmunoResearch Laboratories, Inc). We observed a fluorescent signal around purified dormant spores of both BKH102 and BKH 124 strains (Figure 3). These results indicate that both fusion protein are present on the spore coat surface and are available for antibody binding.

### Efficiency of spore surface display

**Figure 4:**
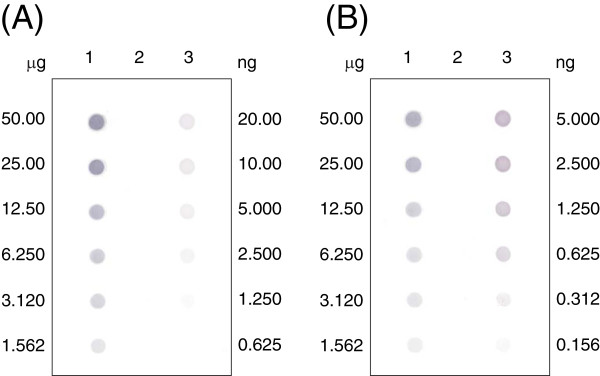
**Dot blot quantification of the amount of fusion proteins in the spore coat. **Panel **A**. Indicated (left) is the concentration of coat proteins isolated from: Lane 1 - BKH102, Lane 2 – 168, Lane – 3 – purified UreA protein, concentration indicated on the right. Panel **B**. Indicated (left) is the concentration of coat proteins isolated from: Lane 1 - BKH124, Lane 2 – 168, Lane – 3 – purified UreA protein, concentration indicated on the right.

For quantitative determination of the amount of CotZ-UreA and CotB-GGGEAAAKGGG-UreA fusion proteins present on the spore coat surface, dot-blotting analysis was used. Serial dilution of purified UreA protein and proteins extracted from BKH102 or BKH124 spore coats were prepared. The UreA, CotZ-UreA and CotB-GGGEAAAKGGG-UreA fusions were detected by anti-UreA antibodies, followed by anti-mouse antibodies with alkaline phosphatase. The colour reaction was performed with the NBT/BCIP system and densitometric analysis using Quantity-One software (Bio-Rad) as described in Methods. As shown in Figure 4, the results revealed that the fusion protein CotZ-UreA constituted 0.02% of total spore coat protein from strain BKH102 (Table 2). From these results, we calculated that the number of fusion protein molecules extracted from a single spore was 1.4 × 10^2^. In the case of CotB-GGGEAAAKGGG-UreA we calculated that the fusion constituted 1.1% of total spore coat proteins, which translates into 10^4^ molecules of fusion protein per single dormant spore (Table 2).

**Table 2:** Densitometric analysis

**UreA source**	**Amount of protein used (ng)**	**Density in OD/mm**^**2 **^**(standard deviation)**	**UreA concentration (ng) in extracts (% of total)**	**n° of recombinant molecules extracted from each spore**
**Fusion 1**				
Purified UreA	25.0 ng	177.8 (± 0.03)	NA	
	12.5 ng	85,7 (± 0.09)	NA	
	6.25 ng	41.6 (± 0.08)	NA	
KH102 (CotZ-UreA)	10.00 μg	17,4 (± 0,03)	2.44 (0.02)	**2.5 × 10**^**2**^
	5.00 μg	8,6 (± 0,01)	1.20 (0.02)	
	2.50 μg	4,4 (± 0,08)	0.61(0.02)	
**Fusion 2**				
Purified UreA	25.0 ng	121.4 (± 0.02)	NA	
	12.5 ng	61,3 (± 0.02)	NA	
	6.25 ng	30.8 (± 0.05)	NA	
KH124 (CotB-linker -UreA	1.250 μg	67.2 (± 0.03)	13.83 (1.10)	**1.0 × 10**^**4**^
	0.625 μg	34.5 (± 0.09)	7.10 (1,14)	
	0.312 μg	15.6 (± 0.08)	3.21 (1.04)	

The average amount of protein extracted from a single spore was 18.6 pg and 20.9 pg for BKH102 and BKH124 respectively.

## Discussion

Various authors have tested the employment of spores as particles for surface display suitable for vaccination [9-12,19], for review see [5-8,13]. In this work we continue the use of subunit A of the urease (UreA) of *Helicobacter acinonychis*[10], which has been used extensively as an antigen able to induce, an immune response [20-24]. In this study we were looking for improvements in spore surface display, and decided to use CotZ as the anchor protein, based on its localisation in the external layer of the outer coat of *B. subtilis* spores [16]. CotZ as the anchor protein allowed us to use full-length UreA, which was not successful when CotB or CotC were chosen [10]. This suggests good perspectives that CotZ may allow the use of large proteins for spore surface display. This ability may be connected with the preserved localization in the outer layer of the spore – with minimal disturbance of the outer layers caused by the passenger proteins. In addition, the near-surface localisation of the fusion protein should also improve surface exposure of passenger peptide/proteins, which is an important factor for successful use as an antigen. The densitometric analysis revealed that an estimated 1.4 × 10^2^ molecules of the CotB fusion were extracted from each purified spore. In comparison to our previous construct, CotB-UreA1 (1.1 × 10^3^) [10], CotZ-UreA is less abundant. Previous studies have shown that the entire UreA protein as passenger was also produced successfully when fused with CotG. However, unfortunately, the fusion was not localised on the spore surface [10].

The design of a fusion protein is always associated with some risk of failure in obtaining a functional construct. Some approaches are successful, but most of them are not, with the resulting proteins poorly expressed or displayed. The use of a peptide linker, which separates the components of fusions, may help to overcome some of these problems. As a first attempt, a short GGGGS linker was introduced. Unfortunately this modification resulted in loss of surface display of CotB-GGGGS-UreA protein. This might have been a result of introduction of a fragment without any secondary structure, which destabilised whole protein. We obtained promising results in experiments with the linker containing the EAAAK motif, which forms a stable alpha-helical structure [17]. Incorporation of such a linker between fusion partners resulted in improved expression and permitted for efficient surface display of the CotB-GGGEAAAKGGG-UreA protein.

The results presented here showed that we found CotZ is potentially useful for spore surface display in addition to the previously used CotB, CotC and CotG proteins. Immunological experiment showed that CotZ-UreA fusions are the most efficient in stimulating an immunological response in comparison to other antigens in the mouse model [unpublished data].

The presence of any fusion protein may change the structure of the coat. However, the resistance properties of spores carrying proteins successfully displayed on the coat appear to be indistinguishable from the wild type ones at least in laboratory conditions tested. This gives a credit to use recombinant spores as vaccine vehicles in animal or human trials since the passage through the stomach environment should not affect their ability to stimulate immunological response.

Finally, in the case of difficulties with expression, stability or spore surface display of a given fusion we encourage others to use the -GGGEAAAKGGG- peptide linker, which may help to overcome these problems. The relatively short length of the linker makes it easy in use. It can be simply added in frame with the appropriate primer sequence used for PCR amplification of anchor or passenger protein.

## Methods

### Bacterial strains and transformation

*Bacillus subtilis* strains used in this study are 168 (wild type), BKH102 (*amyE::cotZ-ureA*), BKH119 (*amyE::cotB-GGGGS-ureA*) and BKH124 (*amyE::cotB-GGGEAAAKGGG-ureA*) (Table 3). Plasmid amplifications for nucleotide sequencing and subcloning experiments were performed with *Escherichia coli* strain DH5α [25]. Bacterial strains were transformed by previously described procedures: CaCl_2_-mediated transformation of *E. coli* competent cells [22] and transformation of *B. subtilis*[26].

**Table 3:** Strain list

**Strain**	**Relevant genotype**	**Source**
**168**	*trpC2*	[27]
**BKH102**	*amyE::cotZ-ureA*	This work
**BKH119**	*amyE::cotB-ggggs-ureA*	This work
**BKH124**	*amyE::cotB-gggeaaakggg-ureA*	This work

### Construction of gene fusions

DNA coding for the CotYZ coat proteins was PCR amplified using the *B. subtilis* chromosome as a template and oligonucleotide pair cotY-F and cotZ-R (Table 1) as primers for fusion *cotZ*-*ureA*. The amplification product of 1256 bp (*cotZ*-*ureA*) was obtained and cloned into the pDL vector (Bacillus Genetic Stock Center) yielding pDL-CotZ (see Figure 1E).

For the gene fusion with CotB, DNA was amplified using the *B. subtilis* chromosome as a template and oligonucleotide pairs Link-gggs-F and Link-gggs-R (Table 1) as primers for the CotB-GGGS-UreA fusion. In the case of CotB-GGGEAAAKGGG-UreA, fusion linker-gggeaaakggg-F and linker-gggeaaakggg-R were used (Table 1).

A 748 bp DNA fragment coding for UreA was PCR amplified using the *H. acinonychis* chromosome as a template and oligonucleotides ureA-F and ureA-R as the primers (Table 1). The PCR product was sequentially digested with *Bam*HI and *Sac*I and cloned in frame to the 3’ end of the *cotZ* or *cotB* gene carried by plasmid pDL-CotZ, yielding plasmid pKH102, pKH119 or pKH124 respectively.

### Chromosomal integration

Appropriate plasmids were linearized by digestion with a single cutting restriction enzyme. Linearized DNA was used to transform competent cells of the *B. subtilis* strain 168 [28]. Chloramphenicol-resistant (Cm^R^) clones were the result of a double-crossover recombination event, resulting in the interruption of the non-essential *amyE* gene on the *B. subtilis* chromosome. Several Cm^R^ clones were tested by PCR using chromosomal DNA as a template and oligonucleotides AmyS and AmyA [27] to prime the reaction. Selected clones were called BKH102, BKH119 or BKH124 and kept for further studies.

### Preparation of spores

Sporulation was induced by the exhaustion method in DS (Difco-Sporulation) medium as described elsewhere [29]. Sporulating cultures were harvested 24 h after the initiation of sporulation and purified using a lysozyme treatment to break any residual sporangial cells followed by washing steps in 1 M NaCl, 1 M KCl and water (two-times), as described previously [29]. PMSF (0.05 M) was included to inhibit proteolysis. After the final suspension in water spores were treated at 65°C for 1 h to kill any residual cells. The spore suspension was titrated immediately for CFU/ml before freezing at −20°C. By this method we could reliably produce 6 × 10^10^ spores per litre of DSM culture.

### Extraction of spore coat proteins

Spore coat proteins were extracted from 50 μl of a suspensions of spores at high density (1 × 10^10^ spores per ml) using a decoating extraction buffer as described elsewhere [30]. Extracted proteins were assessed for integrity by SDS-polyacrylamide gel electrophoresis (PAGE) and for concentration by two independent methods: the Pierce BCA Protein Assay (Pierce) and the BioRad DC Protein Assay kit (Bio-Rad).

### Western and dot blotting analyses

Extracted proteins were separated in 12% denaturing polyacrylamide gels, electro transferred onto the nitrocellulose filter Roti-NC (ROTH) and used for Western blotting analysis by standard procedures. Western blotting filters were visualized by developing with nitroblue tetrazolium–5-bromo-4-chloro-3-indolylphosphate according to the manufacturer’s instructions. Serial dilutions of extracted proteins and purified UreA were used for dot blotting analysis. Filters were then visualized by incubation with nitroblue tetrazolium–5-bromo-4-chloro-3-indolylphosphate, followed by densitometric analysis with Chemidoc XRS (Bio-Rad) and the MultiAnalyst software.

### Immunofluorescence microscopy

Samples were fixed directly in the medium as described by Harry et al., [31], with the following modifications: after suspension in GTE-lysozyme (50 mM glucose, 20 mM Tris- HCl [pH 7.5], 10 mM EDTA, 2 mg of lysozyme/ml), samples (30 μl) were immediately applied to a microscope slide previously coated with 0.01% (wt/vol) poly-L-lysine (Sigma). After 3 min, liquid was removed and the microscope slide was allowed to dry (2 h at room temperature). The microscope slides were washed three times in phosphate-buffered saline (PBS) (pH 7.4), blocked for 30 min with 3% milk in PBS at room temperature and then washed nine more times with PBS. Samples were incubated overnight at 4°C with polyclonal anti-UreA antibody (raised in mouse), washed ten times and then incubated with anti-mouse IgG-Cy3 conjugates with Cyanine (Jackson ImmunoResearch Laboratories, Inc.) for 2 h at room temperature. After ten washes the coverslip was mounted onto a microscope slide and viewed using a Zeiss Axioplan fluorescence microscope with the same exposure time for all samples. Images were captured using a camera connected to the microscope, processed with Corel Photo-Paint software and saved in TIFF format.

## Competing interests

The authors declare that they have no competing interests.

## Authors’ contributions

KH – performed most of the experiments, AI – contributed discussions and manuscript writing, MO – contributed discussions during the work and wrote most of the manuscript. All authors read and approved the final manuscript.
